# Changes in the symptom pattern and the densities of large-intestinal endocrine cells following *Campylobacter* infection in irritable bowel syndrome: a case report

**DOI:** 10.1186/1756-0500-6-391

**Published:** 2013-09-29

**Authors:** Magdy El-Salhy, Tarek Mazzawi, Doris Gundersen, Jan G Hatlebakk, Trygve Hausken

**Affiliations:** 1 Department of Medicine, Section for Gastroenterology, Stord Helse-Fonna Hospital, Stord, Norway; 2 Department of Research, Helse-Fonna, Haugesund, Norway; 3 Institute of Medicine, Section for Gastroenterology, University of Bergen, Bergen, Norway

**Keywords:** *Campylobacter*, Irritable bowel syndrome, Peptide YY, Quality of life, Serotonin, Somatostatin

## Abstract

**Background:**

Irritable bowel syndrome (IBS) is a common chronic functional gastrointestinal disorder. Post-infectious IBS (PI-IBS) is a subset of IBS that accounts for a large proportion of IBS patients. The PI-IBS symptoms meet the Rome criteria for IBS with diarrhoea (IBS-D) or IBS with mixed bowel habits (IBS-M). A low-grade inflammation has been reported to occur in PI-IBS. Abnormalities in intestinal endocrine cells have been reported in both sporadic IBS and PI-IBS.

**Case presentation:**

A 20-year-old female with a diagnosis of IBS with constipation (IBS-C), according to Rome III criteria, contracted *Campylobacter*-induced gastroenteritis, after which her symptom pattern changed to IBS-M. She showed an intestinal low-grade inflammation that was manifested by an increase in the number of intraepithelial and lamina propria leucocytes and lymphocytes and an increase in the density of mast cells in lamina propria. There was also an increase in the density of intestinal serotonin and peptide YY (PYY) cells and a decrease in the density of rectal somatostatin cells. Follow-up of the patient at 4-months post-infection revealed reduction of IBS symptoms and an improvement in her quality of life. However, 6 months following the *Campylobacter* infection, the patient switched back from IBS-M to IBS-C, probably due to recovery from PI-IBS. The patient was treated with prucalopride, which is serotonin 5HT_4_ receptor agonist. Six months later following this treatment, the symptoms were reduced and the quality of life improved in the reported patient.

**Conclusions:**

Gastroenteritis in patients with IBS-C causes a post-infectious, low-grade inflammation. Interaction between immune-cells and intestinal endocrine cells increases the density of certain endocrine cells, which in turn might be responsible for the change in the symptom pattern, the milder symptoms and the improvement in the quality of life seen in the reported patient. The findings in this case raise the question as to whether intestinal infections are responsible for the previously reported switching of IBS from one subtype to another over time.

## Background

Irritable bowel syndrome (IBS) is a common chronic functional gastrointestinal disorder, that is characterized by frequent abdominal pain/discomfort, abdominal bloating/distension and an altered stool pattern [1-4]. Post-infectious IBS (PI-IBS) is a subset of IBS, and is characterized as a sudden onset of IBS symptoms following gastroenteritis in individuals who have had no gastrointestinal complaints [5]. The proportion of patients developing IBS following gastroenteritis varied between studies, from 3.7% to 36% [5]. Patients with IBS are more common in patients presenting with bacterial gastroenteritis to primary care physician than community controls [6]. This may indicate that IBS patients are predisposed to bacterial gastroenteritis, or that they tend to seek their doctor for bowel symptoms more often than the background population. Human infections caused by *Campylobacter jejuni* are a leading cause of food-borne enteritis, the bacteria usually being transmitted by the ingestion of undercooked poultry, or contact with farm animals. This infection leads to PI-IBS in 9-13% of cases [5,7-9]. The symptoms of PI-IBS meet the Rome criteria for IBS with diarrhoea (IBS-D) or IBS with mixed bowel habits (IBS-M) [10-12].

In IBS, there appears to be a general depletion of gastrointestinal endocrine cells, and especially serotonin and PYY cells [13,14], whereas in PI-IBS there is an increase in the density of these cells, especially serotonin and PYY cells [1,5,11]. Furthermore, a low-grade inflammation has been reported in PI-IBS, which is manifested by increased intraepithelial lymphocytes and an infiltration of mast cells in the lamina propria of the large intestine [5,15-17]. It has been suggested that the alterations in the population of gastrointestinal endocrine cells and the low-grade inflammation play a role in the pathogeneses of both sporadic and PI-IBS [1,5,13].

## Case presentation

A 20-year-old female was investigated for recurrent abdominal pain, abdominal distension, constipation and nausea. She had a bowel movement every 7–10 days, with straining at defecation and hard or lumpy stools. She was non-smoker and was not currently taking any medications. This patient had suffered from these symptoms since her childhood. Her mother had similar symptoms and had a diagnosis of IBS. Her symptoms affected her schoolwork and isolated her socially; she has been hospitalized on many occasions. The patient submitted to a complete physical examination and was investigated by means of blood (full blood count, electrolytes, calcium, and inflammatory markers), liver, and thyroid function tests. She also underwent gastroscopy with duodenal biopsy sampling and colonoscopy with segmental biopsy sampling. The findings of all these examinations and tests were normal. The patient fulfilled Rome III criteria and was thus given the diagnosis of IBS with constipation (IBS-C). She was asked to complete the three following questionnaires (Table 1): Birmingham IBS Symptom scores, Short-Form Nepean Dyspepsia Index (SF-NDI) measuring the reduction in quality of life and Irritable Bowel Syndrome quality of life (IBS-QOL) [18-20]. She was then submitted to a non-pharmacological treatment program at our clinic, which includes provision of information and reassurance, dietary guidance, regular exercise and regular intake of probiotics [21]. Her symptoms subsequently reduced and her quality of life improved.

**Table 1:** **Symptoms and quality of life in the patient before, during and after *Campylobacter *infection**

**Questionnaire**	**Before infection**	**During infection**	**After infection**			
			**2 months**	**4 months**	**6 months**	**12 months**
Birmingham						
Total score	30	35	26	18	29	4
Pain	6	15	4	3	6	2
Diarrhoea	4	20	12	8	5	0
Constipation	20	0	10	7	20	2
SF-NDI	29	48	20	17	28	13
IBS-QOL^a^	61	50	84	86	60	94

Six months after infection, the patient was treated with 2 mg prucalopride daily.

Birmingham, Birmingham Irritable Bowel Syndrome Symptom Questionnaire; SF-NDI, Short-Form Nepean Dyspepsia Index; IBS-QOL, Irritable Bowel Syndrome Quality Of Life Questionnaire.

^a^Percentage of the total score.

Seven months later, the patient was referred to the causal department because of a 3-day history of bloody diarrhoea occurring between 10 to 15 times daily, extreme fatigue and dehydration. She did not have a fever and with the exception of C-reactive protein (CRP), which was 17 mg/l (normal range 0–10 mg/l), her blood tests were normal. Colonoscopy revealed severe colonic inflammation with erythema, oedema, friable mucosae, haemorrhagic spots and ulcers. Biopsy samples taken during colonoscopy revealed preserved crypt architecture. However, a focal increase in the density of immune cells in the lamina propria and focal cryptitis and crypt abscesses were observed. Stool culture was positive for *Campylobacter jujeni.* The patient was treated with 400-mg metronidazole, twice daily for 2 weeks.

The findings of a physical examination and blood tests performed at follow-up visits at the outpatient clinic 2, 4, 6 and 12 months after *Campylobacter* infection were normal. Colonoscopy at 2 and 4 months visits revealed a normal endoscopic appearance. Moreover, the patient’s general condition was improved. Her symptom pattern had changed and she experienced an improvement in her quality of life (Table 1). Reassessment of her symptoms according to Rome III criteria put the patient into the IBS-M subtype. Six months following the *Campylobacter* infection, the patient suffered from abdominal pain, abdominal distension, constipation and nausea in the same degree as before the infection. She was treated with 2 mg prucalopride daily. Six months later, the patient’s symptom was reduced and her quality of life improved (Table 1).

Colonic and rectal biopsy samples obtained during colonoscopy before, during, and 2 and 4 months after *Campylobacter* infection were fixed overnight in 4% buffered paraformaldehyde, embedded in paraffin, and cut into 5-μm sections. The sections were immunostained with the avidin-biotin –complex (ABC) method using Vectastain ABC-kit and 3,3′-diaminobenzidine (DAB) peroxidase Substrate Kit (Vector laboratories). The sections were incubated with the primary antiserum/antibody at room temperature for 2 h. The sections were then washed in PBS buffer and incubated with biotinylated swine anti-mouse (in the case of monoclonal antibodies) or anti-rabbit IgG (in the case of polyclonal antibodies) diluted 1:200 for 30 min at room temperature. After washing the slides in PBS buffer, the sections were incubated for 30 min with avidin-biotin-peroxidase complex diluted 1:100, and then immersed in 3,3′-diaminobenzidine (DAB) peroxidase substrate, followed by counterstaining in hematoxylin. The following primary antisera/antibodies were used: monoclonal mouse anti-N-terminal of purified Chromogranin A (Dako, code no. M869), monoclonal mouse anti-serotonin (Dako, code no. 5HT-209), polyclonal anti-porcine peptide PYY (Alpha-Dagnostica, code PYY 11A), polyclonal rabbit anti-synthetic-human PP (Diagnostic Biosystems, code no. #114), polyclonal rabbit anti-porcine glicentin/glucagon (Acris Antibodies, code BP508), polyclonal rabbit anti-synthetic-human somatostatin (Dako, code no. A566); monoclonal mouse anti-human CD45 (Dako, code no. M0701), monoclonal mouse anti-human CD47 (Dako, code no. I5647), monoclonal mouse anti-human CD68 (Dako, code no. M0814) and monoclonal mouse anti-human mast cell tryptase (Dako, code no. M7052). CD45 is considered as a leucocyte common antigen and is expressed exclusively on cells of the hematopoietic system and their progenitors. CD57 is expressed by subsets of NK cells and CD8+ lymphocytes, and by a small percentage of CD4+/CD45R0+ T lymphocytes. CD68 labels human monocytes, macrophages and myeloid cells. Human mast cell tryptase comprise a family of trypsin-like neutral serine proteases that are predominantly expressed in mast cells. The total leucocytes, lymphocytes and mast cells, as well as chromogranin A, serotonin, peptide YY (PYY), and somatostatin cells. The densities of these cells were quantified by computerized image analysis using Olympus cellSens imaging software (version 1.7) on a computer linked to an Olympus microscope type BX 43 with an Olympus camera (DP 26). A ×40 objective was used, for which each frame (field) on the monitor represented a tissue area of 0.14 mm^2^ of the tissue. The number intraepithelial leucocytes cells and the endocrine cells as well as the area of the epithelial cells were measured in each field. The number of leucocytes, lymphocytes, and mast cells in lamina propria were counted per microscopic field. All measurements were done in 10 randomly chosen fields for each individual.

**Figure 1:**
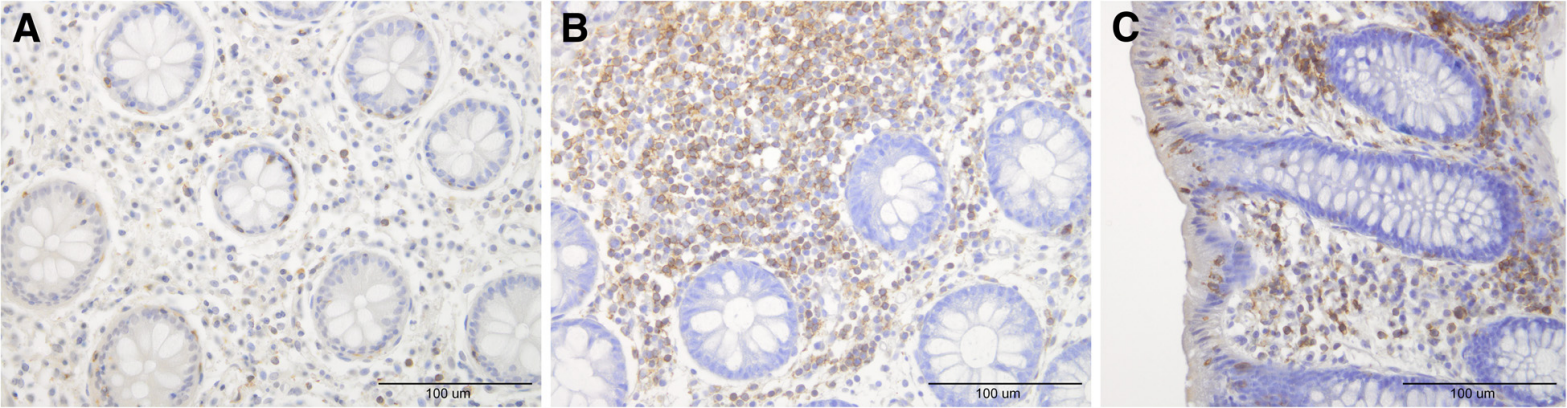
**Leucocytes in the patient before *Campylobacter *infection (A), during (B) and 4-months after (C) *Campylobacter *infection.**

**Figure 2:**
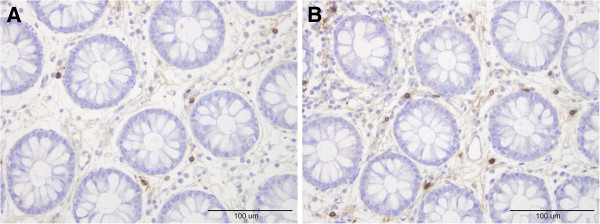
**Mast cells in the lamina propria before *Campylobacter *infection (A) and (B) 4- months after *Campylobacter *infection.**

**Figure 3:**
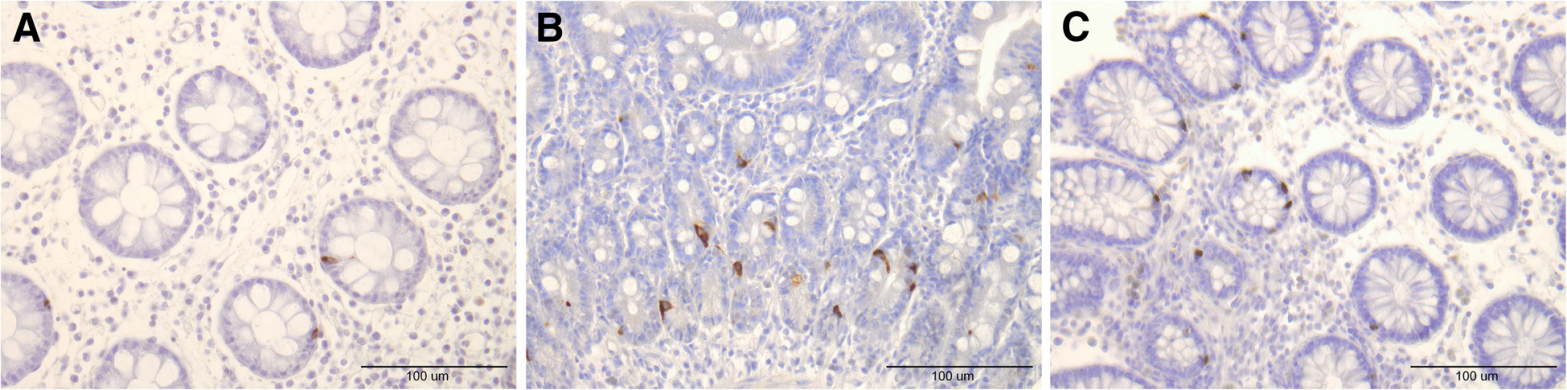
**Serotonin immunoreactive cells before (A), during (B) and 4 months after (C) *Campylobacter *infection.**

The densities of both intraepithelial and lamina propria leucocytes and lymphocytes were increased in both the colon and rectum at 2 and 4 months after the *Campylobacter* infection (Figure 1), as were the number of mast cells in the lamina propria in both the colon and rectum (Figure 2, Tables 2 and 3). The total number of endocrine cells in the colon and rectum prior to *Campylobacter* infection (as detected by chromogranin A staining) was low, but within the normal limits (Tables 4 and 5). This is in agreement with previously published results in IBS-C patients [22,23]. Although chromogranin A is used as a common marker for peptide hormone containing cells, chromogranin A immunoreactivity varies between gastrointestinal segments and even within population of the same endocrine cell type [24]. It has been found that chromogranin A- immunoreactive cells are not representative of the entire population of endocrine cells and that they are the least numerous of all of the endocrine cells combined [25]. The densities of serotonin and PYY cells had increased in both the colon and rectum during, 2 and 4 months post-infection (Figure 3). However, somatostatin cell density in the rectum was reduced in the rectum during and after *Campylobacter* infection.

**Table 2:** **Number of colonic intraepithelial (IE) and lamina propria (LP) immune cells before, during and after *Campylobacter *infection**

**Cell type**	**Before infection**	**During infection**	**After infection**		**Controls**^**c **^**95% confidence interval**
			**2 months**	**4 months**	
Leucocytes in LP^a^	69	268	102	199	81-118
Leucocytes in IE^b^	110	224	162	150	78-115
Lymphocytes in LP^a^	3	39	2	2	0-5
Lymphocytes in IE^b^	1	6	7	8	0-2
Mast cells^a^	7	17	11	12	6-10

Quantifications of cells were conducted in ten randomly chosen fields using the Olympus CellSense software.

^a^Number of cells per field.

^b^Number of cells per mm^2^ of epithelium.

^c^The control group comprised 27subjects (16 females and 11 males; mean age 52 years, range 20–69 years) who had submitted to colonoscopy for the following reasons: gastrointestinal bleeding, where the source of bleeding was identified as haemorrhoids (*n*=18), or angiodysplasia (*n*=2), and health worries resulting from a relative being diagnosed with colon carcinoma (*n*=7).

**Table 3:** **Densities of rectal IE and LP immune endocrine cells before, during and after *Campylobacter *infection**

**Cell type**	**Before infection**	**During infection**	**After infection**		**Controls 95% confidence interval**
			**2 months**	**4 months**	
Leucocytes in LP	71	298	104	202	82–112
Leucocytes in IE	105	224	172	153	81–120
Lymphocytes in LP	1	42	2	2	0-6
Lymphocytes in IE	2	7	7	9	0-2
Mast cells	9	19	14	15	9-12

Quantifications and controls are the same as in Table 2.

**Table 4:** Endocrine cell densities in the colon before, during and after *Campylobacter* infection

**Cell type**	**Before infection**	**During infection**	**After infection**		**Controls 95% confidence interval**
			**2 months**	**4 months**	
Chromogranin A	7	59	50	20	32-43
Serotonin	5	32	32	28	27-32
PYY	4	29	20	15	6-10

Quantifications and controls are the same as in Table 2.

**Table 5:** Densities of rectal endocrine cells before, during and after *Campylobacter* infection

**Cell type**	**Before infection**	**During infection**	**After infection**		**Controls 95% confidence interval**
			**2 months**	**4 months**	
Chromogranin A	35	154	50	65	108–136
Serotonin	21	83	32	43	32–51
PYY	16	49	24	29	54–67
Somatostatin	22	9	3	15	14–20

Quantifications and controls are the same as in Table 2.

## Discussion

Consistent with previously published observations, the present case developed a low-grad inflammation following *Campylobacter* infection [1,5,11,16,26-31]. An increase in the densities of intestinal endocrine cells, and especially serotonin and PYY cells, has been reported in Crohn’s disease, ulcerative colitis and lymphocytic colitis [32-35]. An increase in the density of intestinal endocrine has also been described in PI-IBS [5,11,15,16,28,30,31,33]. Several studies have shown that inflammation and immune cells affect the neuroendocrine system of the gut (the endocrine/immune axis) [1,36]. It seems that infection/inflammation induces an increase in the population of certain gut endocrine cells through an interaction between those cells and immune cells [1,36].

The pattern of symptoms in the present patient changed from IBS-C to IBS-M with much less abdominal pain. Serotonin activates the submucosal sensory branch of the enteric nervous system, and controls gastrointestinal motility and chloride secretion *via* inter-neurons and motor neurons [13,37-42]. PYY delays gastric emptying, inhibits gastric and pancreatic secretion, and is a major ileal brake mediator [13,43,44]. Moreover, PYY inhibits prostaglandin (PG) E2 and vasoactive intestinal peptide (VIP), both of which stimulate intestinal secretion [13,45-47]. Administration of PYY inhibits diarrhoea in experimental animals by reducing intestinal fluid secretion and slowing colon transit [13,48]. Somatostatin inhibits intestinal contraction, and inhibits gut exocrine and neuroendocrine secretion [13]. It is therefore conceivable, that the changes in the present patient’s symptoms are attributable to the reported changes in the density of the endocrine cells.

It is not uncommon for IBS patients to switch from one subtype to another over time [49-52]. The patient presented here switched from the IBS-C subtype to the IBS-M subtype following a bout of gastroenteritis, and it is possible that intestinal infection was the underlying cause of this switch. However, 6 months following the *Campylobacter* infection, the patient switched back from IBS-M to IBS-C. *Campylobacter jejuni* produces a range of toxins including cytolethal distending toxin (24), which first produces secretory diarrhoea in the small intestine early in the illness, after which there is invasion of the distal ileum and colon to produce an inflammatory ileocolitis, which can extend all the way to the rectum [53]. It has been reported that PI-IBS symptoms following *Campylobacter* infection decline with time [54-56]. It is conceivable, therefore, to conclude that the patient returning to her original symptoms represent a recovering form PI-IBS.

The symptoms were reduced and the quality of life improved in the patient following the treatment with prucalopride, which is a highly selective serotonin 5HT_4_ receptor agonist that has been shown to stimulate gut motility [57]. The patient disclosed a low density of colonic serotonin cells, which is in line with previously published observations in IBS patients [14]. This may explain why a serotonin agonist was effective in the treatment of the reported patient.

## Conclusions

Gastroenteritis due to *Campylobacter* infection in patients with IBS-C causes low-grade inflammation and changes in the densities of intestinal endocrine cells. These changes may be responsible for the change in symptom pattern and the switch from IBS-C to IBS-M that were observed in the reported patient. The patient switched back to IBS-C, 6 months following the *Campylobacter* infection, probably as a recovery from IP-IBS. Furthermore, treatment with serotonin agonist was successful in the reported patient, who disclosed reduced colonic serotonin cell density.

## Consent

Written informed consent was obtained from the patient for publication of this Case report and any accompanying images. A copy of the written consent is available for review by the Editor of this journal.

## Competing interests

The authors declare that they have no competing interests.

## Authors’ contributions

ME planned the study, recruited and followed-up the patients, performed three of the four colonoscopies, quantified the immune and endocrine cells, analysed the data and drafted the manuscript. TM contributed to patient follow-up, performed one of the four colonoscopies, contributed to the data analysis of and writing this manuscript. DG contributed to the data analysis and writing this manuscript. JGH checked the data, reviewed the manuscript and contributed to discussions. TH checked the data, reviewed the manuscript and contributed to discussions. All of the authors read and approved the final version of this manuscript.
